# Xiao-Ai-Ping, a TCM Injection, Enhances the Antigrowth Effects of Cisplatin on Lewis Lung Cancer Cells through Promoting the Infiltration and Function of CD8^+^ T Lymphocytes

**DOI:** 10.1155/2013/879512

**Published:** 2013-07-17

**Authors:** Wanshuai Li, Yang Yang, Zijun Ouyang, Qi Zhang, Lu Wang, Feifei Tao, Yongqian Shu, Yanhong Gu, Qiang Xu, Yang Sun

**Affiliations:** ^1^State Key Laboratory of Pharmaceutical Biotechnology, School of Life Sciences, Nanjing University, 22 Hankou Road, Nanjing 210093, China; ^2^Department of Clinical Oncology, The First Affiliated Hospital of Nanjing Medical University, 140 Hanzhong Road, Nanjing 210029, China

## Abstract

*Objectives*. To investigate how Xiao-Ai-Ping injection, a traditional Chinese medicine and an ancillary drug in tumor treatment, enhances the antitumor effects of cisplatin on Lewis lung cancer (LLC) cells. *Methods*. LLC-bearing mice were daily intraperitoneally injected with various doses of cisplatin, Xiao-Ai-Ping, or cisplatin plus Xiao-Ai-Ping, respectively. Body weight and tumor volumes were measured every three days. *Results*. Combination of Xiao-Ai-Ping and cisplatin yielded significantly better antigrowth and proapoptotic effects on LLC xenografts than sole drug treatment did. In addition, we found that Xiao-Ai-Ping triggered the infiltration of CD8^+^ T cells, a group of cytotoxic T cells, to LLC xenografts. Furthermore, the mRNA levels of interferon-**γ** (*ifn-* **γ**), perforin-1 (*prf-1*), and granzyme B (*gzmb*) in CD8^+^ T cells were significantly increased after combination treatment of Xiao-Ai-Ping and cisplatin. *In vitro* studies showed that Xiao-Ai-Ping markedly upregulated the mRNA levels of *ifn-* **γ**, *prf-1,* and *gzmb* in CD8^+^ T cells in a concentration-dependent manner, suggesting that Xiao-Ai-Ping augments the function of CD8^+^ T cells. *Conclusions*. Xiao-Ai-Ping promotes the infiltration and function of CD8^+^ T cells and thus enhances the antigrowth effects of cisplatin on LLC xenografts, which provides new evidence for the combination of Xiao-Ai-Ping and cisplatin in clinic in China.

## 1. Introduction

Innate and adaptive immunities play important roles in the development and progression of cancer. It is becoming apparent that tumors can influence the induction of potentially protective responses in a number of ways [1]. Immunotherapies which induce immune responses to tumors have been investigated for over 100 years as an attractive strategy for cancer treatment [2, 3]. Cancer chemotherapy drugs have long been considered immune suppressive. However, more recent data indicate that some cytotoxic drugs effectively treat cancer in part by facilitating an immune response to the tumor when given at the standard dose and schedule [2]. More recently, two clinical trials of antibodies which target the negative immune checkpoint molecule PD-1 on T cells and its ligand B7-H1/PD-L1 on tumor cells were unexpectedly successful, with durable response rates of 20–25% in advanced melanoma, renal cell cancer, and nonsmall-cell lung cancer (NSCLC) [2, 4, 5], suggesting the critical role of enhancing immunity in cancer therapy. Immune-mediated restriction of tumor growth requires synchronization of several interdependent events, including activation of tolerized immune cells [6, 7]. Effector CD8^+^ T cells occupy a key position in cancer immunotherapeutic approaches [6, 8]. CD8^+^ cytotoxic T cells recognize tumor antigens and kill tumor cells essentially though the release of interferon-*γ* (IFN-*γ*), perforin, and granzymes [8–11]. 

Chemotherapy is one of the key tools used in tumor treatment [12–14], and whether chemotherapy drugs promote antitumor immunity is one of the crucial factors that determine the effects of the drugs [1, 2, 15–17]. Plant-derived natural products play an important role in cancer chemotherapy. Numerous molecules, such as paclitaxel, vincristine, camptothecin, and epipodophyllotoxin, are invaluable contributions of nature to modern medicine [18]. In recent years, interest in using natural products as therapeutic agents for cancer has sharply increased [12, 19–23]. Thus, the quest to search for novel therapeutic compounds or strategies for cancer treatment and management is a never-ending venture.

*Marsdenia tenacissima*, the stem of *Marsdenia tenacissima* (*Roxb*.) *Wight et Arn*. (Asclepiadaceae Family), is widely grown in the southern provinces of China [12]. More than 40 C-21 steroidal glycosides have been isolated from *Marsdenia tenacissima* [24], some of which were proved to show cytotoxic activities against cancer cells [25]. Xiao-Ai-Ping injection, a standard *M. tenacissima* extract, is shown to be clinically effective in treatment of NSCLC when combined with chemotherapeutics agents [26–28]. However, the exact mechanism of the combination strategy remains vague. In the present study, we demonstrate that Xiao-Ai-Ping promotes the infiltration and function of CD8^+^ cytotoxic T cells and thus enhances the antigrowth effects of cisplatin on mouse Lewis lung cancer cells. Our data provide the molecular theoretical basis for clinical application of Xiao-Ai-Ping injection in patients with NSCLC.

## 2. Materials and Methods

### 2.1. Cells and Reagents

Lewis lung cancer (LLC) cells were purchased from the Shanghai Institute of Cell Biology (Shanghai, China), maintained in Dulbecco Minimum Essential Medium (DMEM, GIBCO) supplemented with 10% fetal bovine serum (FBS, GIBCO), 100 U/mL penicillin, and 100 *μ*g/mL streptomycin and incubated at 37°C in a humidified atmosphere containing 5% CO_2_ in the air. Xiao-Ai-Ping injection (5 g crude/mL) was provided by Sanhome Pharmaceutical Co., Ltd (Nanjing, China) and stored at 4°C. Cisplatin was purchased from Sigma Aldrich Chemical Co. (St. Louis, MO). For *in vivo* experiments, Xiao-Ai-Ping and cisplatin were diluted in phosphate buffered saline (PBS); for *in vitro *experiments, Xiao-Ai-Ping was diluted in fresh media. Phospho-Akt (Thr 308) (#2965) antibody was purchased from Cell Signaling Technology (Beverly, MA). PCNA (#sc-7907), and GADD153/CHOP (#sc-793) antibodies were purchased from Santa Cruz Biotechnology (Santa Cruz, CA). Granzyme B antibody (#6632-1) was purchased from Epitomics (Burlingame, CA). All other chemicals were purchased from Sigma Aldrich Chemical Co. (St. Louis, MO).

### 2.2. Animals

C57BL/6J mice (6 to 8 weeks old) were purchased from the Shanghai Laboratory Animal Center (Shanghai, China). Briefly, mice were fed with free access to pellet food and water in plastic cages at 21 ± 2°C and kept on a 12-hour light-dark cycle. Animal welfare and experimental procedures were carried out strictly in accordance with the Guide for the Care and Use of Laboratory Animals (The Ministry of Science and Technology of China, 2006) and the related ethical regulations of our university. All efforts were made to minimize animals' suffering and to reduce the number of animals used.

### 2.3. RNA Extraction, Reverse Transcription Polymerase Chain Reaction (RT-PCR), and Quantitative PCR (Q-PCR) Analysis

Total RNA extraction and RT-PCR were performed as published before [29]. Briefly, cells were collected and lysed in Trizol (Invitrogen). One *μ*g extracted RNA was used for reverse transcription with Oligo(dT) primers (Takara). For RT-PCR analysis, 1 *μ*L cDNA products were used, and the primers were as follows: *ifn-*γ** Sense (5′−3′): ATGAACGCTACACACTGCATC, Antisense (5′−3′): CCATCCTTTTGCCAGTTCCTC; *prf-1 *Sense (5′−3′): AGCACAAGTTCGTGCCAGG, Anti-sense (5′−3′): GCGTCTCTCATTAGGGAGTTTTT; *gzmb *Sense (5′−3′): CCACTCTCGACCCTACATGG, Anti-sense (5′−3′): GGCCCCCAAAGTGACATTTATT; *rn18s *Sense (5′−3′): AGTCCCTGCCCTTTGTACACA, Anti-sense (5′−3′): CGATCCGAGGGCCTCACTA. All primers were purchased from GenScript (Nanjing, China). cDNA amplification was performed for 30 cycles with the following settings: 94°C for 5 min, 94°C for 30 sec, 58°C for 30 sec, and 72°C for 30 sec, and 72°C for 10 min. The RT-PCR products were electrophoresed on a 2% agarose gel and visualized by ethidium bromide staining. The Gel Imaging and Documentation DigiDoc-It System (version 1.1.23; UVP, Inc., Upland, CA) was used to scan the gels. 18S ribosome RNA (*Rn18s*) was performed as a loading control. For Q-PCR analysis, the abundance of gene expression was analyzed with SsoFast EvaGreen Supermix (Bio-Rad) on CFX96 Touch Real-Time PCR Detection System (Bio-Rad) and normalized with *rn18s* level.

### 2.4. TUNEL and Immunohistochemistry Staining

TUNEL staining was performed using TUNEL FITC Apoptosis Detection Kit from Vazyme Biotech Co., Ltd (Nanjing, China) according to the manufacturer's protocols. Immunohistochemistry staining was performed using GT Vision III Immunohistochemistry Detection Kit (GK500710) from GeneTech Company Limited (Shanghai, China) according to the manufacturer's protocols. The fluorescent signals were detected with a mercury lamp (Olympus U-RFL-T) and analyzed by Image-Pro Plus 6.0 (Media Cybernetics, Inc. Bethesda, MD, USA). 

### 2.5. *In Vivo* Tumor Growth Assay

LLC cells were cultured in 100 mm dishes (Costar) and collected in 50 mL centrifuge tubes. Then the cells were centrifuged (1000 rpm, 5 min) and washed twice with ice-cold PBS. The cells were diluted to 1 × 10^6^/mL before injection. 1 × 10^6^ LLC cells (in 0.1 mL PBS) were injected subcutaneously into the right flanks of female C57BL6/J mice (6–8 weeks of age). All mice formed tumors three days after injection. Then mice were distributed into four groups (*n* = 8) according to tumor volumes, and the day was marked as day 0. Vehicle and indicated doses of drugs were administered to every group respectively every day since day 0. Tumor length and tumor width were measured with a vernier caliper every three days. Tumor volumes were calculated according to the following formula: 0.5 × tumor length × tumor width^2^. Tumor weight, body weight, and spleen weight were measured after the mice were euthanized. Tumor sections were infused in formaldehyde solution for immunohistochemistry. The rest sections were shortly frozen in liquid nitrogen and then stored at −80°C.

### 2.6. Preparation of Mouse CD8^+^ T Cells

Mouse CD8^+^ T cells were isolated from the lymph nodes with Dynabeads FlowComp Mouse CD8 Kit (11462D, Invitrogen) according to the manufacturer's protocols. Briefly, 1 mL (1 × 10^8^) cells isolated from the lymph nodes were transferred to a tube and incubated with 50 *μ*L FlowComp Mouse CD8 antibody for 10 min at 4°C. The cells were resuspended in 2 mL isolation buffer (Ca^2+^ and Mg^2+^ free PBS supplemented with 0.1% BSA and 2 mM EDTA) after being washed once with 4 mL isolation buffer. Then the suspensions were incubated with 150 *μ*L washed FlowComp Dynabeads for 15 min at 4°C under rolling and tilting. Then 2 mL isolation buffer was added, and the tube was placed in a magnet for 2 min. The supernatant containing CD8 negative cells was carefully removed and discarded, while the tube was still in the magnet. The bead-bound CD8^+^ cells were washed once with isolation buffer. Then CD8^+^ cells were released from the beads with release buffer (modified biotin in 0.1% BSA and 2 mM EDTA). The cells were kept at 4°C until further use. 

### 2.7. Statistical Analysis

Data are expressed as means ± SD. One way ANOVA was used to test for differences among more than two groups. The Student's *t*-test was used to evaluate the difference between two groups. *P* < 0.05 was considered to be significant.

## 3. Results

### 3.1. Cisplatin Dose-Dependently Inhibited the Growth of LLC Xenografts While Xiao-Ai-Ping Had Little Effect on the Growth of LLC Xenografts

**Figure 1:**
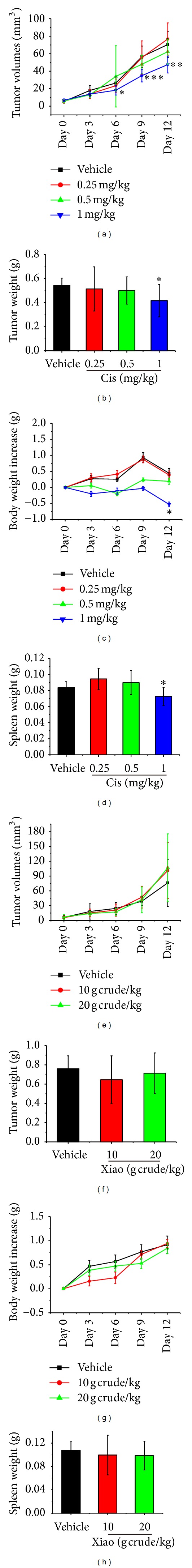
Cisplatin dose-dependently inhibited the growth of LLC xenografts while Xiao-Ai-Ping had little effect. Various doses of cisplatin (Cis, 0.25, 0.5, 1 mg/kg, *n* = 8 per group) or Xiao-Ai-Ping (Xiao, 10, 20 g crude/kg *n* = 8 per group) were daily i.p. injected to LLC-bearing mice, respectively, for 12 days. Body weight and tumor volumes were measured every three days. (a–d) Tumor volumes (a), tumor weight (b), body weight (c), and spleen weight (d) of cisplatin-treated LLC-bearing mice. (e–h) Tumor volumes (e), tumor weight (f), body weight (g), and spleen weight (h) of Xiao-Ai-Ping treated LLC-bearing mice. *P < 0.05, **P < 0.01, ***P < 0.001 versus vehicle (PBS). LLC, Lewis lung cancer.

To examine the respective effects of cisplatin and Xiao-Ai-Ping on the growth of LLC xenografts, various doses of cisplatin (0, 0.25, 0.5, 1 mg/kg) and Xiao-Ai-Ping (0, 10, 20 g crude/kg, the doses were determined according to the clinical dosage of Xiao-Ai-Ping injection) were, respectively, intraperitoneally (i.p.) injected to the LLC-bearing mice for 12 days. One mg/kg cisplatin led to a significant decrease in the growth and weight of LLC xenografts (Figures 1(a) and 1(b)), while 20 g crude/kg Xiao-Ai-Ping only had little effects (Figures 1(e) and 1(f)). It was also shown that 1 mg/kg cisplatin significantly decreased the body weight and spleen weight of LLC-bearing mice (Figures 1(c) and 1(d)), while 20 g crude/kg  Xiao-Ai-Ping had no influences (Figures 1(g) and 1(h)). Considering the clinical dosage of Xiao-Ai-Ping injection, we use 20 g crude/kg  Xiao-Ai-Ping combined with 1 mg/kg cisplatin in the following study.

### 3.2. Xiao-Ai-Ping Promoted the Antigrowth Effect of Cisplatin on LLC Xenograft

**Figure 2:**
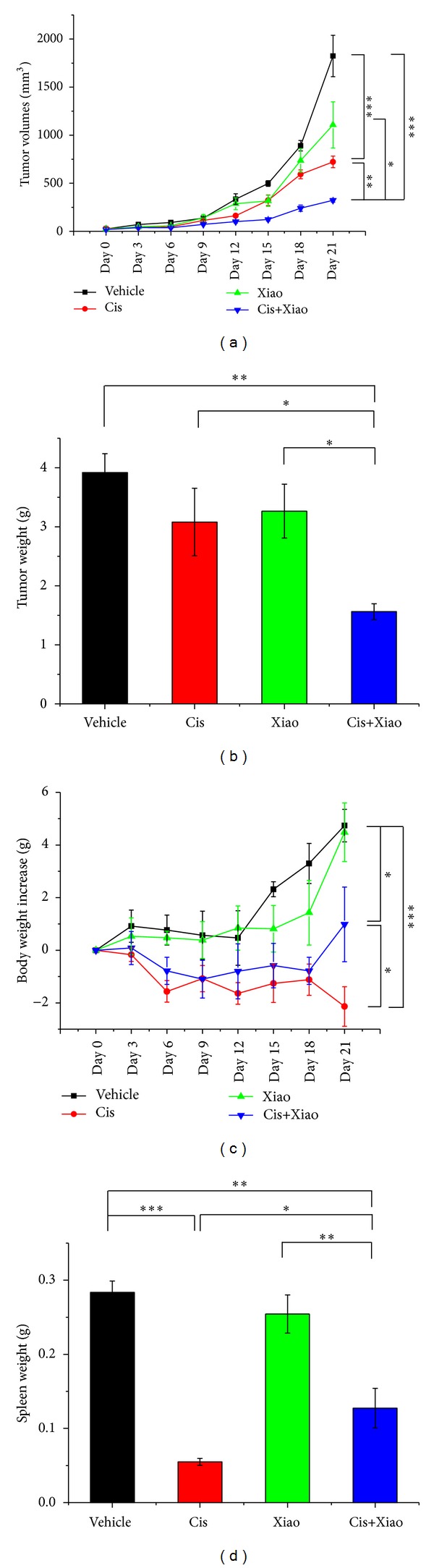
Combination of cisplatin with Xiao-Ai-Ping had significantly better effects than cisplatin. PBS (Vehicle, *n* = 8), 1 mg/kg cisplatin (Cis, *n* = 8), 20 g crude/kg Xiao-Ai-Ping (Xiao, *n* = 8), and 1 mg/kg cisplatin plus 20 g crude/kg Xiao-Ai-Ping (Cis+Xiao, *n* = 8) were daily i.p. injected for 21 days, respectively. (a) Tumor volumes. (b) Tumor weight. (c) Body weight. (d) Spleen weight. *P < 0.05, **P < 0.01, ***P < 0.001.

Vehicle (PBS, *n* = 8), cisplatin (1 mg/kg, *n* = 8), Xiao-Ai-Ping (20 g crude/kg, *n* = 8), and cisplatin plus Xiao-Ai-Ping (1 mg/kg cisplatin plus 20 g crude/kg Xiao-Ai-Ping, *n* = 8) were i.p. injected into LLC-bearing mice for 21 days. Cisplatin (1 mg/kg) significantly reduced the volumes (*P* < 0.001, Figure 2(a), the red line) and weight (*P* < 0.05, Figure 2(b), the red column) of LLC xenografts, while 20 g crude/kg Xiao-Ai-Ping only had slight effects (no significant difference versus vehicle group, Figure 2(a), the green line and Figure 2(b), the green column). Interestingly, combination of 1 mg/kg cisplatin with 20 g crude/kg Xiao-Ai-Ping had significantly better effects than 1 mg/kg cisplatin (Figure 2(a), the blue line and Figure 2(b), the blue column). It should be noted that 1 mg/kg cisplatin significantly decreased the body weight (*P* < 0.001, Figure 2(c), the red line) and spleen weight (*P* < 0.001, Figure 2(d), the red column) of LLC-bearing mice. However, Xiao-Ai-Ping, at the dose of 20 g crude/kg, had no effects on the body weight (Figure 2(c), the green line and Figure 2(d), the green column) or spleen weight (Figure 2(d), the green column) of LLC-bearing mice. Notably, combination of cisplatin with Xiao-Ai-Ping remarkably attenuated the toxicity of cisplatin on the body weight (Figure 2(c), the blue line) and spleen weight (Figure 2(d), the blue column). These results imply that Xiao-Ai-Ping enhances the antigrowth effects of cisplatin on LLC xenografts and attenuates the side effects of cisplatin.

### 3.3. Xiao-Ai-Ping Enhanced the Proapoptotic Effect of Cisplatin on LLC Xenografts

**Figure 3:**
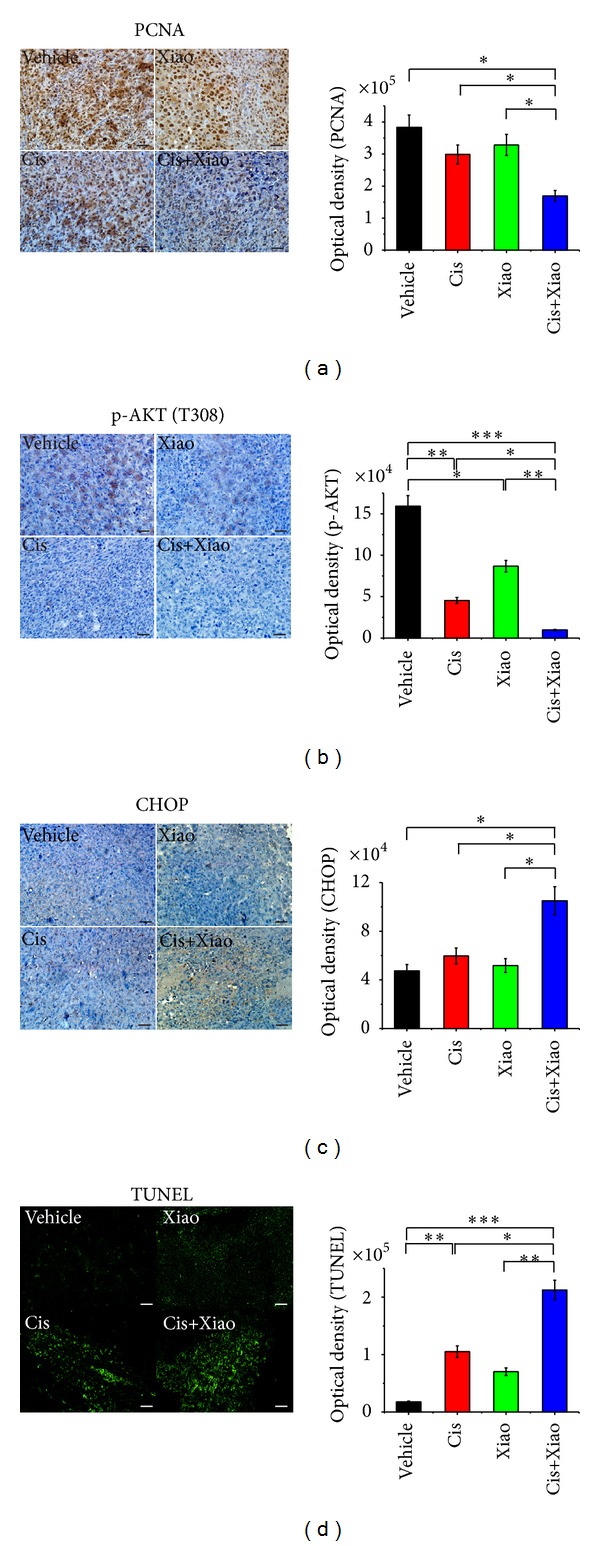
Xiao-Ai-Ping promotes the antigrowth and proapoptotic effects of cisplatin on LLC xenografts. PBS (Vehicle, *n* = 8), 1 mg/kg cisplatin (Cis, *n* = 8), 20 g crude/kg Xiao-Ai-Ping (Xiao, *n* = 8), and 1 mg/kg cisplatin plus 20 g crude/kg Xiao-Ai-Ping (Cis+Xiao, *n* = 8) were daily injected for 21 days, respectively. Tumor sections were infused in formaldehyde solution for immunohistochemistry. (a) Expression of PCNA (left panel) and average optical density of five samples (right panel). (b) Expression of phospho-Akt (left panel) and average optical density of five samples (right panel). (c) Expression of CHOP (left panel) and average optical density of five samples (right panel). (d) TUNEL staining of tumor sections (left panel) and average optical density of five samples (right panel). Images were representative of eight samples per group, and quantitation data were average of eight samples. Scale bars, 10 *μ*m. *P < 0.05, **P < 0.01, ***P < 0.001.

Anti-growth and proapoptotic effects of chemotherapy drugs are crucial for cancer treatment [30, 31]. Here we detected the levels of several markers for tumor growth and apoptosis. Proliferating cell nuclear antigen (PCNA) and phosphorylation of protein kinase B (Akt) are markers for tumor growth [32, 33]. DNA damage-inducible transcript 3, also known as C/EBP homologous protein (CHOP), is a key marker for tumor apoptosis [34, 35]. Compared with sole drug treatment, 1 mg/kg cisplatin combined with 20 g crude/kg Xiao-Ai-Ping significantly decreased the protein levels of PCNA (Figure 3(a)) and phosphorylated Akt (Thr 308) (Figure 3(b)) in LLC xenografts. Meanwhile, the protein level of CHOP was significantly increased (Figure 3(c)). TUNEL staining implied that 1 mg/kg cisplatin combined with 20 g crude/kg Xiao-Ai-Ping induced significantly more apoptosis than sole drug treatment did (Figure 3(d)).

### 3.4. Xiao-Ai-Ping Increased the Infiltration and Function of CD8^+^ T Cells

**Figure 4:**
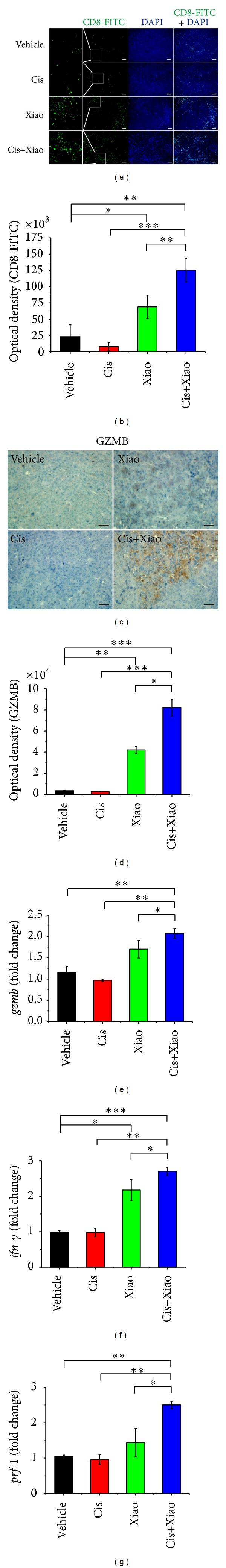
Xiao-Ai-Ping enhances infiltration and functions of CD8^+^ T cells in LLC xenografts. PBS (Vehicle, *n* = 8), 1 mg/kg cisplatin (Cis, *n* = 8), 20 g crude/kg Xiao-Ai-Ping (Xiao, *n* = 8), and 1 mg/kg cisplatin plus 20 g crude/kg Xiao-Ai-Ping (Cis+Xiao, *n* = 8) were daily i.p. injected for 21 days, respectively. Tumor sections were infused in formaldehyde solution for immunofluorescence or shortly frozen in liquid nitrogen for RNA extraction. (a and b) (a) Immunofluorescence of tumor sections. Green, CD8-FITC (Invitrogen); blue, DAPI (4,6-diamidino-2-phenylindole, Sigma). Scale bars, 20 *μ*m. Images were representative of eight samples per group. (b) Quantitation of CD8-FITC (average of eight samples per group). (c and d) Immunohistochemisty stain of tumor sections. (c) Representative images of eight samples per group. Scale bars, 10 *μ*m. (d) Quantitation of GZMB (average of eight samples per group). (e–g) The mRNA levels of *gzmb *(e)*, ifn-*γ** (f), and *prf-1* (g) were analyzed by Q-PCR and normalized with *rn18s*. *P < 0.05, **P < 0.01, ***P < 0.001.

CD8^+^ T cells play an important role in anti-tumor immunity [6, 36, 37]. Release of interferon-*γ* (IFN-*γ*), perforin, and granzymes is one crucial way of CD8^+^ cytotoxic T cells to kill tumor cells [6, 37, 38]. In the following study, we found that combination of 1 mg/kg cisplatin with 20 g crude/kg Xiao-Ai-Ping significantly increased the infiltration of CD8^+^ T cells to LLC xenografts (*P* < 0.01, Figure 4(a) and Figure 4(b)). Meanwhile, mRNA and protein level of granzyme B (*P* < 0.05, Figures 4(c)–4(e)), and mRNA levels of *ifn-*γ** (Figure 4(f)) and *prf-1* (Figure 4(g)) were significantly increased in cisplatin-plus-Xiao-Ai-Ping-treated xenografts, compared to cisplatin-treated or Xiao-Ai-Ping-treated xenografts.

**Figure 5:**
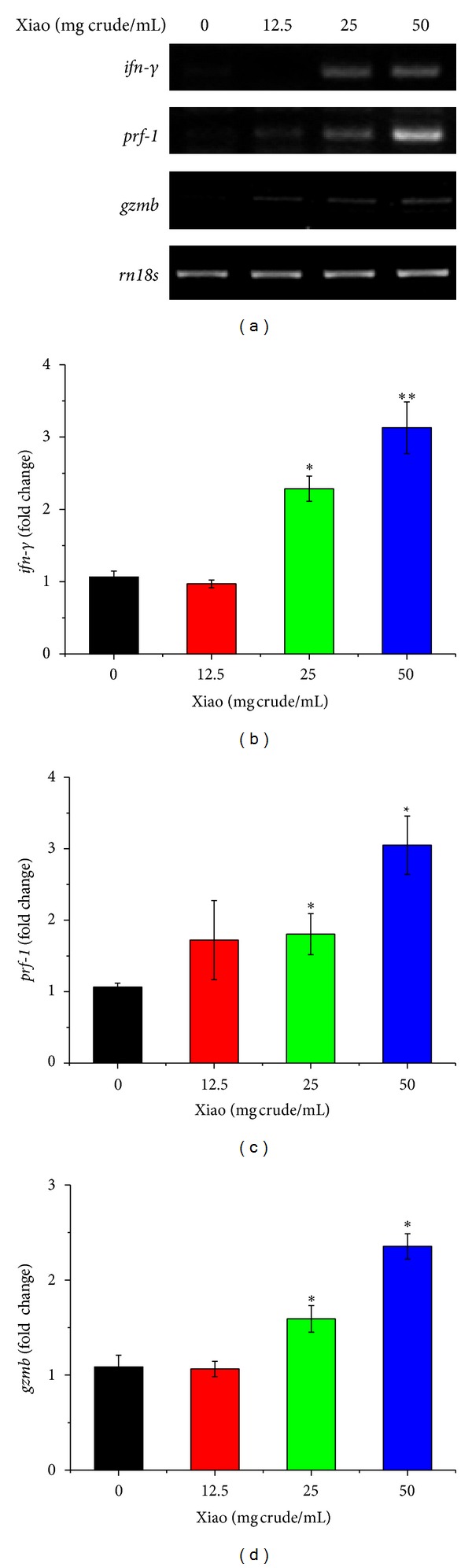
Xiao-Ai-Ping increases the mRNA expressions of *ifn-*γ**, *prf-1,* and *gzmb* of CD8^+^ T cells *in vitro*. CD8^+^ T cells isolated from the lymph nodes of female C57BL6/J mice were incubated with various concentrations of Xiao-Ai-Ping (Xiao) for 12 h. Total RNA from the cells were extracted. (a) RT-PCR analysis of *ifn-*γ**, *prf-1,* and *gzmb*. *Rn18s* was performed as a loading control. All the experiments were repeated at least three times, and one representative result was shown. (b–d) mRNA expression of *ifn-*γ** (b), *prf-1* (c), and *gzmb* (d), normalized with *rn18s*. *P < 0.05, **P < 0.01 versus 0 mg crude/mL Xiao-Ai-Ping. All the experiments were repeated at least three times.

In the *in vitro* study, CD8^+^ T cells were isolated from mouse lymph nodes and incubated with various concentrations (0, 12.5, 25, and 50 mg crude/mL) of Xiao-Ai-Ping for 12 h. Results showed that Xiao-Ai-Ping significantly enhanced the mRNA levels of *ifn-*γ**, *prf-1*, and *gzmb* in a concentration-dependent manner (Figure 5). Considering that herbal extracts tend to be contaminated with lipopolysaccharide (LPS) during preparation, we detected the concentration of LPS in Xiao-Ai-Ping injection with Tachypleus Amebocyte Lysate kit (Chinese Horseshoe Crab Reagent manufactory Co., Ltd., Xiamen, China). No LPS contamination was detected in Xiao-Ai-Ping injection. Therefore, the changes in mRNA levels of *ifn-*γ**, *prf-1*, and *gzmb *were not caused by contaminated LPS.

**Figure 6:**
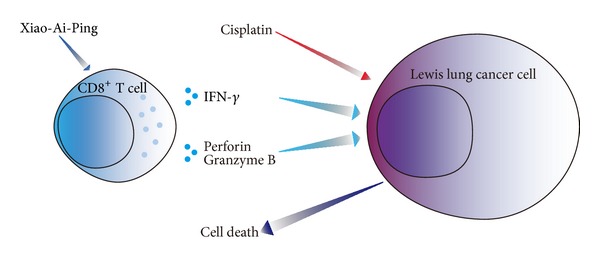
Overview of mechanism of synergetic anticancer effect of combination of cisplatin with Xiao-Ai-Ping on Lewis lung cancer cells. Xiao-Ai-Ping promotes the release of IFN-*γ*, perforin, and granzyme B from CD8^+^ T cells, promotes the functions of CD8^+^ cytotoxic T cells and thus enhances the antigrowth and proapoptotic effects of cisplatin on LLC cells.

In conclusion, as described in Figure 6, these results suggest that Xiao-Ai-Ping promotes the infiltration of CD8^+^ cytotoxic T cells to the tumor site and enhances the expressions of IFN-*γ*, perforin, and granzyme B from CD8^+^ T cells and thus facilitates the antigrowth and proapoptotic effects of cisplatin.

## 4. Discussion

Chemotherapy combined with immunotherapy has been an effective strategy for cancer treatment [1–3, 6]. In our present study, we demonstrated that traditional Chinese medicine Xiao-Ai-Ping injection enhanced the anti-growth and pro-apoptotic effects of cisplatin on LLC xenografts through promoting the infiltration and function of CD8^+^ cytotoxic T cells. 

It should be noted that Xiao-Ai-Ping only had slight effects on growth and apoptosis of LLC xenografts. However, combination of cisplatin with Xiao-Ai-Ping induced significantly more apoptosis of LLC xenografts than cisplatin alone did. To our interest, the effect of 1 mg/kg cisplatin alone was not satisfactory, and Xiao-Ai-Ping alone only had slight antitumor effects. So we wondered why combination of cisplatin with Xiao-Ai-Ping had such great synergetic anti-tumor effects. 

In the *in vitro* study, we found that Xiao-Ai-Ping dose-dependently enhanced the function of CD8^+^ T cells, as the mRNA levels of *ifn-*γ**, *prf-1*, and *gzmb* of T cells were increased by the treatment of Xiao-Ai-Ping. Moreover, Xiao-Ai-Ping remarkably increased the infiltration and function of CD8^+^ T cells in LLC xenografts. Meanwhile, combination of cisplatin with Xiao-Ai-Ping induced much more infiltration of CD8^+^ T cells in LLC xenografts. Cisplatin did not promote either infiltration or function of CD8^+^ T cells, despite its strong anti-tumor effect. These results suggest that cisplatin and Xiao-Ai-Ping might play different roles against cancer. Cisplatin induced apoptosis of LLC cells and directly killed cancer cells, while Xiao-Ai-Ping regulated the immune system of LLC-bearing mice and promoted anticancer immune response. Notably, low dose of cisplatin (1 mg/kg) only induced limited apoptosis of LLC cells but greatly suppressed the immune system of LLC-bearing mice, which restricted its anticancer effect and clinical dosage. Most importantly, when cisplatin was combined with Xiao-Ai-Ping, the weights of body and spleen from LLC-bearing mice were no longer decreased so drastically as cisplatin sole group. In addition, more infiltration and function of CD8^+^ T cells were seen in the combination group than in the sole one. From the data of Figure 2, we know that Xiao-Ai-Ping alone had slight anticancer effect on LLC xenografts. In the process of killing LLC cells, cisplatin exerts a dominant role, while Xiao-Ai-Ping plays a supportive function. Xiao-Ai-Ping's auxiliary role is essential to achieve better anti-LLC effects of cisplatin, since cisplatin usually impairs the anti-tumor immunity in tumor-bearing mice. Xiao-Ai-Ping alone hardly kills LLC cells since Xiao-Ai-Ping has little cytotoxic effects, although Xiao-Ai-Ping increases the infiltration and function of CD8^+^ T cells. These results collectively suggest that Xiao-Ai-Ping promotes the anticancer effect of cisplatin by enhancing anticancer immunity in mice. 

## 5. Conclusions

The current study demonstrates for the first time that Xiao-Ai-Ping promotes the infiltration and function of CD8^+^ cytotoxic T cells and modulates the immune system in tumor-bearing mice and thus enhances the anticancer effect of cisplatin. The present study gives a theoretical support for the clinical use of Xiao-Ai-Ping injection in patients with NSCLC.
